# TRAIL Mediates Neuronal Death in AUD: A Link between Neuroinflammation and Neurodegeneration

**DOI:** 10.3390/ijms22052547

**Published:** 2021-03-04

**Authors:** Liya Qin, Jian Zou, Alexandra Barnett, Ryan P. Vetreno, Fulton T. Crews, Leon G. Coleman

**Affiliations:** 1 Bowles Center for Alcohol Studies, School of Medicine, The University of North Carolina at Chapel Hill, Chapel Hill, NC 27599, USA; ambarn@live.unc.edu; 2 Department of Psychiatry, School of Medicine, The University of North Carolina at Chapel Hill, Chapel Hill, NC 27599, USA; fulton_crews@med.unc.edu; 3 Department of Pharmacology, School of Medicine, The University of North Carolina at Chapel Hill, Chapel Hill, NC 27599, USA

**Keywords:** neurodegeneration, toll-like receptor 7, apoptosis, TRAIL, TNFSF10, death receptors

## Abstract

Although the cause of progressive neurodegeneration is often unclear, neuronal death can occur through several mechanisms. In conditions such as Alzheimer’s or alcohol use disorder (AUD), Toll-like receptor (TLR) induction is observed with neurodegeneration. However, links between TLR activation and neurodegeneration are lacking. We report a role of apoptotic neuronal death in AUD through TLR7-mediated induction of death receptor signaling. In postmortem human cortex, a two-fold increase in apoptotic terminal deoxynucleotidyl transferase dUTP nick end labeling (TUNEL) staining in neurons was found in AUD versus controls. This occurred with the increased expression of TLR7 and tumor necrosis factor (TNF)-related apoptosis-inducing ligand (TRAIL) death receptors. Binge ethanol treatment in C57BL/6 mice increased TLR7 and induced neuronal apoptosis in cortical regions that was blocked by TLR7 antagonism. Mechanistic studies in primary organotypic brain slice culture (OBSC) found that the inhibition of TLR7 and its endogenous ligand let-7b blocked ethanol-induced neuronal cell death. Both IMQ and ethanol induced the expression of TRAIL and its death receptor. In addition, TRAIL-neutralizing monoclonal antibodies blocked both imiquimod (IMQ) and ethanol induced neuronal death. These findings implicate TRAIL as a mediator of neuronal apoptosis downstream of TLR7 activation. TLR7 and neuronal apoptosis are implicated in other neurodegenerative diseases, including Alzheimer’s disease. Therefore, TRAIL may represent a therapeutic target to slow neurodegeneration in multiple diseases.

## 1. Introduction

Neuronal cell death can occur through multiple mechanisms in settings of trauma, ischemia, or disease [1,2]. In settings that involve progressive neurodegeneration such as alcohol use disorder (AUD), aging, or Alzheimer’s disease, the underlying cell death mechanisms can be more difficult to identify. Progressive pathologic neurodegeneration is often associated with a concomitant increased expression of neuroimmune genes and signs of microglial activation [3,4]. This can involve Toll-like receptor (TLR) activation through endogenous agonists known as damage-associated molecular pattern molecules (DAMPs) [4,5,6]. In diseases with a neurodegenerative component, concurrent increases in TLR signaling are usually observed [7,8,9]; however, the contribution of immune activation to ongoing neurodegeneration is often unclear. 

TLR7 senses single-stranded (ss)RNAs, and it has been found to recognize certain endogenous miRNAs, such as miRNA let-7b, resulting in caspase-mediated neuronal cell death [10,11,12]. Studies suggest that this mechanism may play a role in neurodegenerative diseases. We have reported that TLR7 might contribute to neurodegeneration with alcohol abuse, with ethanol increasing the expression of TLR7 along with secretion of the endogenous agonist miRNA let-7b in primary ex vivo organotypic brain slice culture (OBSC) [10,13]. Let-7 isoforms are increased in postmortem AUD brain and in brain of mice after chronic ethanol treatment [14,15], and TLR7 agonism was recently found to increase alcohol self-administration [16]. Increased levels of let-7 have also been reported in the CSF of Alzheimer’s patients, suggesting that let-7/TLR7 signaling may cross multiple neurodegenerative disorder [17]. 

Regarding alcohol-induced neuronal death, we and others have reported that frank necrosis can contribute to neuronal death in rodent models of very heavy binge exposure [18,19]. However, recent work suggests that apoptotic mechanisms also contribute. We recently reported an increase in apoptotic death receptor (DR) signaling machinery in postmortem human AUD brain [20]. This included increases in DR adapter molecules such as fas-associated protein with death domain (FADD) and tumor necrosis factor receptor type 1-associated death domain protein (TRADD) as well as downstream caspases in human postmortem AUD brain [20]. Regulators of these extrinsic cell death cascade mediators are also altered in Alzheimer’s [21] and Parkinson’s [22] diseases. DR signaling in the periphery is often coupled to innate immune activation [23], although a connection to TLR signaling in brain has not been reported. TLR7-mediated neurodegeneration has been shown to involve the activation of capsase-3 [11]. Thus, we hypothesized that TLR7 might contribute to AUD neurodegeneration through the induction of extrinsic apoptotic death receptor cascades. 

Tumor necrosis factor (TNF)-related apoptosis-inducing ligand (TRAIL or TNFSF10) is a TNF-superfamily DR ligand that activates DR4 (TRAIL-R1) and DR5 (TRAIL-R2) to cause apoptosis through their intracellular adapter protein FADD to induce caspase activation [23,24,25]. TRAIL is an interface between immunity and apoptosis, as it can cause either immune activation or cell death in different settings [26,27]. TRAIL can cause neuronal cell death in human brain tissue [28] and cultured neurons [29]; however, a role for TRAIL in TLR-mediated neurodegeneration has not been reported to date. TRAIL has been linked to TLR7-mediated cell death in response to HIV in peripheral immune cells [30]. Therefore, we investigated if neurodegeneration in AUD involves a TLR7 induction of TRAIL-induced apoptosis. 

In AUD, neuronal loss increases with duration of alcohol abuse and is found in regions such as the orbitofrontal cortex (OFC) [31,32,33], entorhinal cortex (ENT) [19,34], superior frontal gyrus [35], and hippocampus [36,37,38]. Neuronal loss in the OFC is associated with cognitive impairment to promote increased risk of relapse [33,39], poor decision making [32], memory deficits [36], and loss of cognitive control [40,41]. Damage to the ENT involves learning and memory deficits through its regulation of hippocampal function [18,19,38,42,43]. We found increased neuronal apoptosis by apoptotic terminal deoxynucleotidyl transferase dUTP nick end labeling (TUNEL) staining in postmortem human AUD OFC. This was accompanied by induction of TRAIL DRs, executioner caspase-3 activation, and TLR7 protein expression. TLR7 protein in human OFC was positively correlated with lifetime alcohol consumption, which is consistent with a progressive enhancement of signaling across the disease course. In a model of chronic binge ethanol in vivo, we found an induction of apoptosis in OFC and ENT along with increased TLR7 protein expression in neurons, and increased expression of the TLR7 agonist miRNA let-7b. TLR7 induced by ethanol was functionally active, with binge ethanol pretreatment enhancing neurodegeneration induced by the TLR7 agonist imiquimod (IMQ). Ethanol-induced neuronal death was blocked by the pharmacological inhibition of TLR7, implicating TLR7 in ethanol-induced neurodegeneration. Mechanistic studies in primary ex vivo OBSC confirmed that let-7/TLR7 signaling mediates ethanol-induced neuronal cell death and found that this involves the induction of TRAIL and its death receptors, as TRAIL-neutralizing antibodies prevented neurodegeneration due to both IMQ and ethanol. Together, these studies implicate TLR7/TRAIL signaling in AUD neurodegeneration. Given the involvement of TLR signaling in multiple neurodegenerative diseases, let-7/TLR7/TRAIL signaling may represent a fundamental mechanism for neurodegeneration linked to neuroimmune activation that could be targeted therapeutically. 

## 2. Results 

### 2.1. Induction of Neuronal Apoptosis, TRAIL Death Receptors, and TLR7 in Orbitofrontal Cortex (OFC) of Human Subjects with AUD

**Figure 1:**
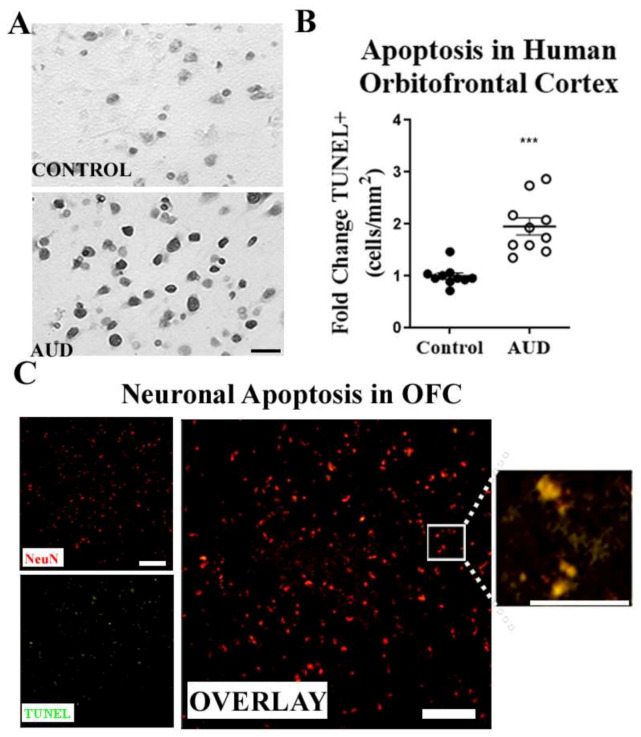
Induction of apoptotic neuronal cell death in human AUD orbitofrontal cortex. Paraffin-embedded sections from human postmortem orbitofrontal cortex (OFC) of moderate drinking controls and individuals with alcohol use disorder (AUD) were assessed for apoptotic cell death by terminal deoxynucleotidyl transferase dUTP nick end labeling (TUNEL) staining. Scale bar = 10 µm. (**A**) Representative image of increased TUNEL+ cells with darker staining in AUD OFC. (**B**) Apoptotic TUNEL+ cells were counted per mm^2^ and fold change was measured relative to controls. The number of TUNEL+ apoptotic cells was increased from baseline levels by 2-fold in human AUD OFC compared to age-matched controls. *** *p* < 0.001. *n* = 10 per group, paired t-tests. (**C**) Immunofluorescence of neuronal marker NeuN (red), TUNEL (green). Overlay shows co-localization of NeuN and TUNEL stain (yellow). High magnification image of selected area (white box) on right panel illustrates co-localization of TUNEL and NeuN. Scale bar = 100 µm.

**Figure 2:**
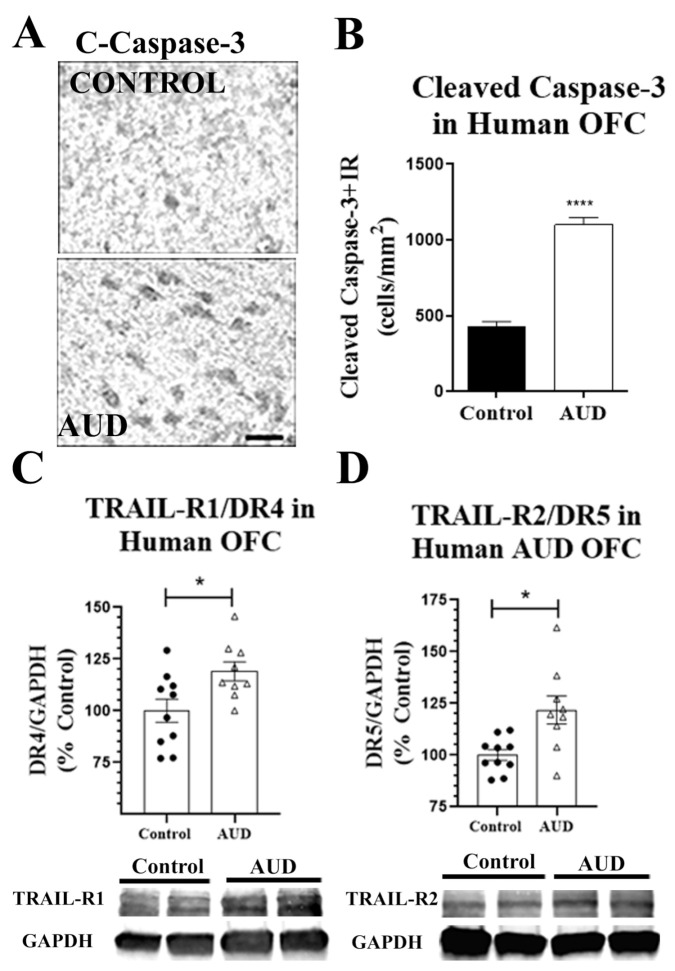
Apoptotic executioner caspase-3 and tumor necrosis factor (TNF)-related apoptosis-inducing ligand (TRAIL) receptors are increased in the OFC of human subjects with AUD. Postmortem human orbitofrontal cortex (OFC), both paraffin-embedded sections and frozen tissue, were assessed by immunohistochemistry (IHC) and Western blot, respectively. (**A**) Representative image of activated, cleaved caspase-3 in postmortem human OFC. (**B**) Quantification of activated, cleaved caspase-3 found a 2-fold increase in cleaved caspase-3 +IR cells consistent with cell death. **** *p* < 0.0001 vs. control, *n* = 10 per group, paired t-tests. (**C**) TRAIL death receptor TRAIL-R1/DR4 was increased by 20% in AUD subjects. (**D**) TRAIL death receptor TRAIL-R2/DR5 was increased by 21% in AUD subjects. * *p* < 0.05 vs. control.

We used cadaveric sections from the New South Wales Brain Tissue Resource Centre (NSW-BTRC) [44] to determine if neuronal apoptosis contributes to neurodegeneration in AUD. Tissue sections from the orbitofrontal cortex (OFC) from individuals diagnosed with AUD, and moderate drinking controls were matched for both age and postmortem interval (Table 1). We found a robust increase in TUNEL+ apoptotic cells in the OFC of individuals with AUD (Figure 1A,B). Co-immunofluorescent labeling of a fluorescent TUNEL stain and the neuronal marker NeuN confirmed the apoptosis of neurons in AUD (Figure 1C). The expression of molecular mediators of apoptosis was also increased in human AUD OFC. This included the activated (cleaved) form of the executioner caspase-3 and the TRAIL death receptors. A two-fold increase in cleaved-caspase-3 was measured by immunohistochemistry (IHC) in the OFC of AUD subjects (Figure 2A,B, **** *p* < 0.0001). This was accompanied by increased expression of both TRAIL death receptors TRAIL-R1/DR4 and TRAIL-R2/DR5 (≈1.2-fold, * *p* < 0.05, Figure 2C,D). The DR5 protein in OFC was positively correlated with TUNEL+ apoptotic cells (R = 0.61, ** *p* < 0.01), suggesting a functional relationship. To determine if ethanol can directly increase the expression of DR4 and DR5 in human neurons, we treated cultured human SH-SY5Y neurons with ethanol and measured DR4 and DR5 expression by Western blot. Ethanol increased TRAIL-R1/DR4 by 30% and slightly increased TRAIL-R2/DR5 by ≈12% within 12 h of exposure (Figure S1). Thus, these findings implicate apoptosis with induction of TRAIL apoptotic death receptors in neuronal loss in AUD. 

**Figure 3:**
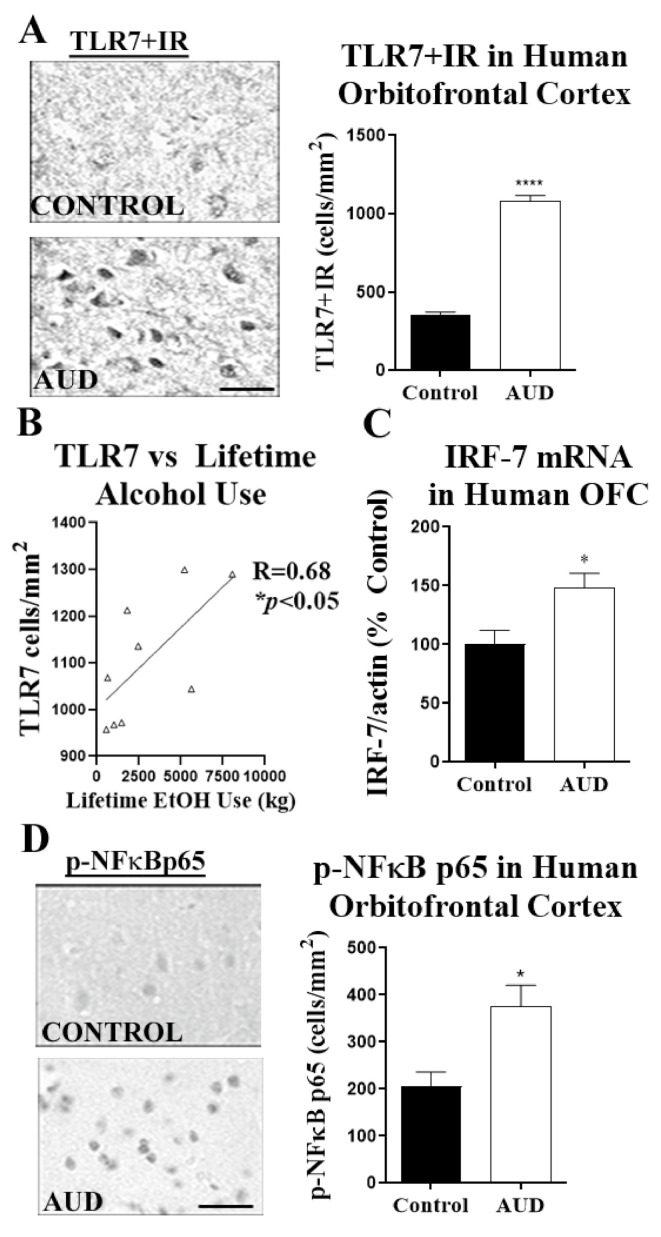
TLR7 protein expression is increased in human AUD OFC and correlates with lifetime consumption of alcohol. (**A**) TLR7+ cell numbers in the OFC were assessed by immunohistochemistry (IHC). A representative image shows a robust increase in TLR7+IR in OFC. TLR7 immunoreactive (+IR) cell counts in OFC were increased by 3-fold in humans with AUD (1079.1 ± 38.3 vs. 354.2 ± 19.6 +IR cells/mm^2^, **** *p* < 0.0001). Scale bar = 10 µm (**B**) TLR7+IR cell numbers from AUD subjects were positively correlated with lifetime consumption of alcohol (EtOH). R = 0.68, * *p* < 0.05. (**C**) Increased expression of the TLR7-associated transcription factor interferon regulatory factor-7 (IRF7) in postmortem human AUD OFC. (**D**) Levels of active phosphorylated nuclear factor kappa-light-chain-enhancer of activated B cell p65 subunit (p-NFκB p65) were measured by IHC. A representative image of p-NFκB p65+IR shows a clear increase in immunoreactive cells in AUD OFC. p-NFκB p65 was increased by 80% (376.3 ± 44.2 vs. 205.2 ± 31.2 +IR cells/mm^2^, * *p* < 0.05). *n* = 9–10 per group, paired t-tests.

Since we found increased apoptosis in postmortem human AUD brain, and TLR7 can cause caspase-mediated neuronal death [10,12] and TRAIL-mediated death of plasmacytoid dendritic cells [30], we assessed if TLR7 was increased in the OFC of human subjects with AUD. In subjects with AUD, we found a 3-fold increase in TLR7 + cells by IHC using our modified stereology approach [45,46,47] (Figure 3A), as well as a 3-fold increase in TLR7 mRNA (* *p* < 0.05). Western blot analysis confirmed an increase in total TLR7 protein (Figure S1A, ≈20%). Among individuals with AUD, the number of TLR7+ cells was positively correlated with lifetime consumption of alcohol (R = 0.68, * *p* < 0.05, Figure 3B). This relationship was not seen with moderate drinking controls. Furthermore, in addition to an increased expression of TLR7, transcription factors downstream of TLR7 were also increased. This included a ≈50% increase in the interferon regulatory factor-7 (IRF7) mRNA (Figure 3C). There was no difference in IRF5 mRNA. In addition, IHC for the transcriptionally active phosphorylated nuclear factor kappa-light-chain-enhancer of activated B cell p65 subunit (pNFκB p65) found an 80% increase in the number of pNFκB p65 immunoreactive cells in human postmortem AUD OFC (Figure 3C, * *p* < 0.05). Western blot also confirmed an increase in total pNFκB p65 protein (Figure S1B, ≈22%). Thus, postmortem human AUD OFC has increased neuronal apoptosis, TRAIL death receptor expression, and induction of TLR7 signaling.

### 2.2. Chronic Binge Ethanol Causes let-7b/TLR7 Signaling and Apoptosis in Cortex

**Figure 4:**
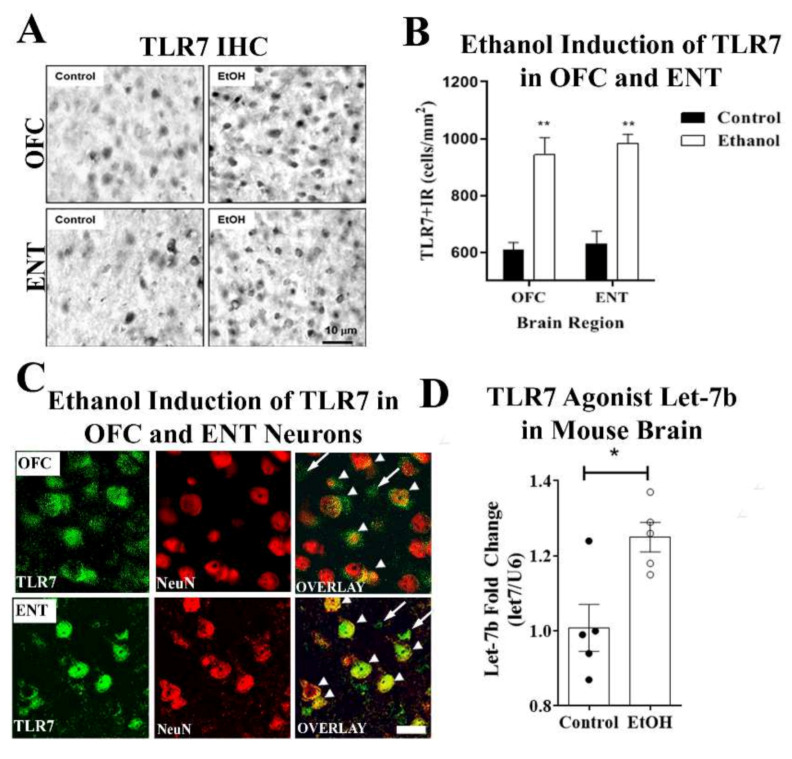
Binge ethanol induces TLR7 protein and miRNA let-7b in mouse cortex. (**A**) C57BL/6 mice were treated with ethanol (5 g/kg, i.g.) once daily for 10 days. Mice were sacrificed 24 h after the last administration and assessed for TLR7 expression in the orbitofrontal (OFC) and entorhinal (ENT) cortices. Representative images of TLR7+IR cells in the OFC and ENT of control and ethanol-treated mouse brain regions. Scale bar = 10 µm. (**B**) Ethanol increased the number of TLR7 immunoreactive (+IR) cells in the OFC and ENT by 1.55- and 1.56-fold, respectively. ** *p* < 0.01, *n* = 8 OFC and *n* = 10 ENT per group. (**C**) Ethanol induction of TLR7 was observed in neurons (arrowheads) as well as in glia (long arrows) in OFC and ENT. Double immunofluorescent confocal images from ENT of a representative ethanol-treated subject. Scale bar = 20 µm. (**D**) C57BL/6 mice were treated with ethanol (5 g/kg, i.g.) once daily for 10 days. Mice were sacrificed 24 h after the last administration and assessed for miRNA let-7b expression and innate immune gene induction by RT-PCR as in Methods. Binge ethanol caused a 25% increase in expression of the endogenous TLR7 agonist miRNA let-7b. * *p* < 0.05.

Since findings in postmortem human AUD brain cannot determine causality, we utilized a chronic 10-day mouse binge-ethanol exposure in which mice receive 5 g/kg/day, i.g., for 10 days. In mice, this dose yields blood alcohol concentrations similar to those found in human binge drinkers, ≈200 mg/dL. Mice were sacrificed 26 h after the last dose, allowing ethanol clearance, which is a time point that we have previously found to have increased neuronal death [34,38,48]. Consistent with findings in human AUD cortex, ethanol caused an increase in TLR7+IHC protein in both OFC and ENT (Figure 4A,B). In order to determine if TLR7 expression was induced in neurons, we performed co-immunofluorescence for TLR7 with neurons (NeuN). Ethanol induced robust co-localization of TLR7 with NeuN+ neurons in both OFC and ENT as well as in smaller non-neuronal cells (Figure 4C). Accordingly, the co-immunofluorescence of TLR7 was also found in Iba-1+ microglia (Figure S2A, top panel) in ENT, with minimal co-localization with GFAP+ astrocytes (Figure S2A, lower panel). The expression of TLRs is often induced by their agonists. Therefore, we measured expression of the endogenous TLR7 agonist, the microRNA let-7b, after chronic binge ethanol. Binge ethanol increased let-7b microRNA (Figure 4D, * *p* < 0.05). In addition, similar to findings in the human AUD cortex, ethanol increased the activation of nuclear factor kappa-light-chain-enhancer of activated B cell (NFκB) with more than a 2-fold increase in the number of pNFκB-p65+IR cells (Figure S2B,C). Furthermore, let-7b expression correlated positively with TLR7 (Figure S2D, * p < 0.02), as well as pro-inflammatory genes across all subjects (i.e., control and ethanol), including interleukin 6 (IL-6) (Figure S2E, * *p* < 0.02), TNFα (Figure S2F, *p* = 0.05), and monocyte chemoattractant protein-1 (MCP-1) (Figure S2G, * *p* < 0.03). Thus, binge ethanol exposure increases brain Let-7/TLR7 signaling and neuroimmune activation, mimicking findings in postmortem human AUD cortex.

### 2.3. Chronic Binge Ethanol Sensitizes TLR7-Mediated Immune Signaling and Promotes Neurodegeneration through TLR7

Recent studies suggest that TLR and innate immune signals in brain are persistently sensitized and progress with additional stimulation [10,34,48,49]. TLR7 can induce both pro-inflammatory activation as well as neuronal death. Therefore, we assessed a role for TLR7 in ethanol-induced immune genes and neurodegeneration. Thus, since chronic 10-day binge ethanol exposure increased TLR7 expression, we hypothesized that it would also sensitize the brain to acute responses to a TLR7 agonist. To determine TLR7 responsiveness, we used a small molecule TLR7 agonist that crosses the blood–brain barrier, imiquimod (IMQ), and compared the responses of mice after binge ethanol exposure (5 g/kg, i.g.,10 days + 24 h) to controls (Figure 4A). We first assessed for acute enhancement of pro-inflammatory gene expression. IMQ alone at this time point (2.5 mg/kg, i.p., 2 h) had no effect on TNFα and increased MCP-1 (2-fold) and IL-1β (4.6-fold, Figure S3). Chronic binge ethanol alone caused increases in mRNA of TNFα (2-fold) and MCP-1 (2.4-fold) but no change in IL-1β. Ethanol pretreatment followed by IMQ caused synergistic increases in the expression of TNFα (7-fold), MCP-1 (10.7-fold), and IL-1β (8.6-fold), which is consistent with sensitization to TLR7 signaling by chronic ethanol. Chronic binge ethanol slightly increased IMQ induction of TLR7 (Figure S4), HMGB1 protein (Figure S5), and microglial activation (Iba-1 IHC, Figure S6). However, IMQ-induced neuronal cell death was also synergistically increased by chronic binge ethanol pretreatment.

Next, we assessed for the role of TLR7 in ethanol-induced neurodegeneration. Fluoro-Jade B, which visualizes actively dying cells, and cleaved caspase-3, the activated executioner caspase, were measured. The 10-day binge ethanol model we employed causes neuronal death in cortical brain regions [35]. 

**Figure 5:**
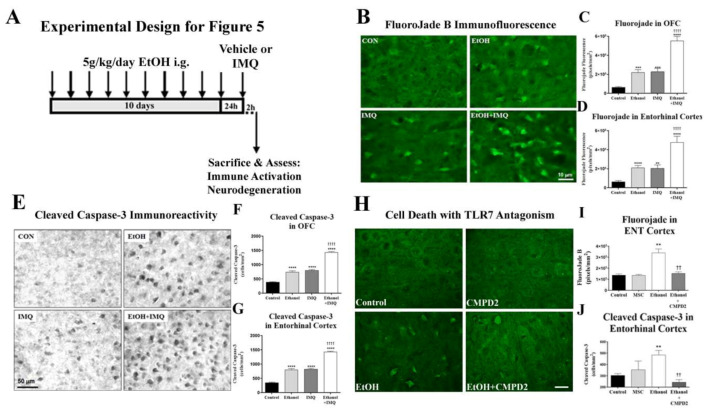
Chronic ethanol causes neuronal death in mouse cortex through TLR7. (**A**) Experimental Design. Mice underwent 10 days of binge ethanol (5 g/kg, i.g.) followed by the TLR7 agonist imiquimod (IMQ, 2.5 mg/kg, i.p.) or vehicle at 24 h after the last ethanol dose. Cell death was assessed in orbitofrontal (OFC) and entorhinal (ENT) cortices by Fluoro-Jade B and cleaved caspase-3 staining. (**B**) Images of cortical Fluoro-Jade B fluorescence show that ethanol pretreatment causes a synergistic increase in IMQ-induced neurodegeneration. (**C**) Quantification in OFC found Fluoro-Jade B fluorescence was increased 3.5-fold by chronic binge ethanol. IMQ alone caused a 3.6-fold increase in Fluoro-Jade B staining. Chronic binge ethanol prior to IMQ caused a synergistic 7.4-fold increase in Fluoro-Jade B. This was 2.4-fold greater than IMQ alone. ANOVA: F3.32 = 37.2, *p* < 0.0001, **** *p* < 0.001 vs. control, †††† *p* < 0.0001 vs. IMQ. (**D**) Quantification in ENT found that Fluoro-Jade B fluorescence was increased 3.3-fold by chronic binge ethanol. IMQ alone caused a 3.2-fold increase in Fluoro-Jade B. Chronic ethanol treatment prior to IMQ resulted in a synergistic 7.4-fold increase in Fluoro-Jade B staining ANOVA: F3.31 = 17.9, *p* < 0.0001, **** *p* < 0.001 vs. control, †††† *p* < 0.0001. (**E**) Representative images of cortical cleaved caspase-3+IR showing chronic binge ethanol enhancement of TLR7-mediated cell death. (**F**) Quantification in OFC found ethanol caused a 1.9-fold increase in cleaved caspase-3. IMQ alone caused a similar 2-fold increase in cleaved caspase-3. Chronic ethanol followed by IMQ 24 h after the last dose resulted in a 3.7-fold increase in cleaved caspase-3+IR ANOVA: F3.38 = 180.0, *p* < 0.0001, **** *p* < 0.001 vs. control, †††† *p* < 0.0001 vs. IMQ. (**G**) Quantification in ENT found that ethanol caused a 2.3-fold increase in cleaved caspase-3. IMQ alone caused a 2.3-fold increase in cleaved caspase-3. Chronic ethanol followed by IMQ 24 h after the last dose resulted in a 4-fold increase in cleaved caspase-3. ANOVA: F3.28 = 207.3, *p* < 0.0001. **** *p* < 0.001 vs. control, †††† *p* < 0.0001 vs. IMQ. *n* = 8–9 mice/group. (**H**) An additional cohort of mice received 10-day binge ethanol ± the TLR7 antagonist CMPD2 (30 mg/kg/day, i.g., 30 min prior to ethanol). Representative images of Fluoro-Jade B stain in the ENT show the blockade of ethanol-induced cell death by CMPD2. (**I**) Quantification in ENT found that Fluoro-Jade B fluorescence was increased ≈3-fold by chronic binge ethanol. CMPD2 prevented the ethanol induction of neuronal death, returning Fluoro-Jade B to control levels. (**J**) Quantification of cleaved caspase-3 staining in ENT found a ≈3-fold increase by chronic binge ethanol. CMPD2 prevented the ethanol induction of neuronal death, returning cleaved caspase-3 to control levels. ** *p* < 0.01 vs. control, †† *p* < 0.01 vs. ethanol, *n* = 4–5/group.

Chronic binge ethanol alone and IMQ alone similarly increased Fluoro-Jade B (≈3.4-fold, Figure 5A–C) and cleaved caspase-3+IR (≈2-fold, Figure 5D,F) in OFC and ENT. However, IMQ following chronic binge ethanol exposure increased both cell death markers 7- to 8-fold in OFC and ENT (**** *p* < 0.0001, Figure 5B,C). Thus, ethanol sensitizes TLR7-induced neurodegeneration in addition to neuroimmune activation. To determine if TLR7 blockade prevents ethanol neurodegeneration, we used CMPD2, a small molecule TLR7 antagonist [50]. CMPD2 given 30 min prior to each ethanol dose completely prevented neuronal death in the ENT (Figure 5G–I). These findings, as well as findings in postmortem human AUD, implicate that binge ethanol induces TLR7 and sensitizes its activation, resulting in increased neuroimmune activation and neurodegeneration. 

**Figure 6:**
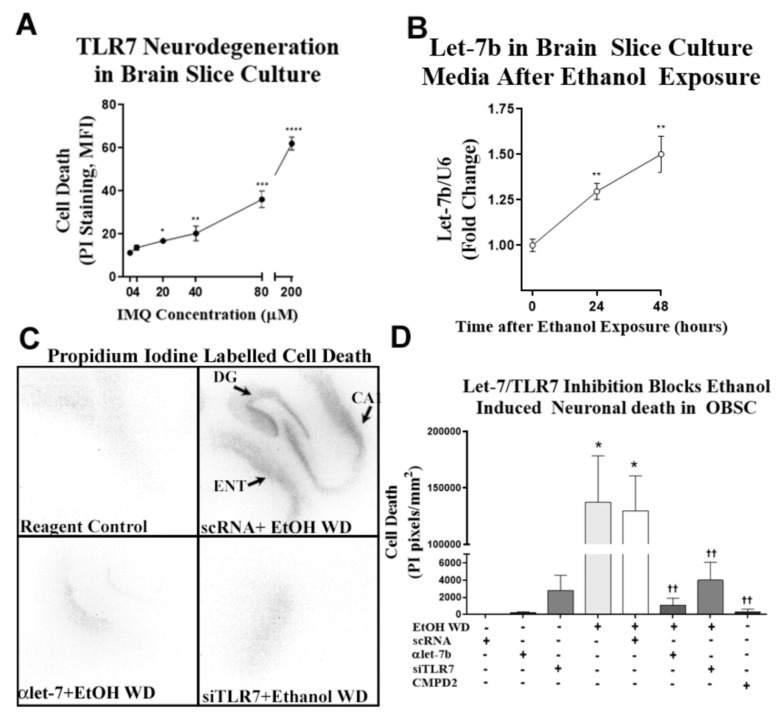
Chronic ethanol causes neuronal death in mouse cortex through TLR7. (**A**) The TLR7 agonist imiquimod (IMQ, 72 h) induces neurodegeneration in organotypic brain slice culture (OBSC) in a concentration-dependent fashion. (**B**) Ex vivo OBSC was treated with ethanol (100 mM) for 4 days, followed by a 24- to 48-h ethanol-free period to induce neurodegeneration. Let-7b was measured in media microvesicles at 24 and 48 h after ethanol withdrawal. Let-7b secretion into the media increased progressively during withdrawal reaching significance at 24 h (30% increase) and at 48 h (50% increase) ** *p* < 0.01. (**C**) Representative images of propidium iodide (PI) staining showing neurodegeneration in sections containing entorhinal cortex (ENT), dentate gyrus (DG) and cornu ammonis (CA) regions of OBSC after EtOH WD. Fluorescent PI images were converted to black and white images for ease of visualization. Both the let-7 antagomir (αlet-7) and siRNA against TLR7 (siTLR7) returned PI uptake to control levels. (**D**) Inhibition of let-7b or TLR7 prevented ethanol-induced neurodegeneration. After ethanol treatment, media was replaced with ethanol-free media and either anti-let-7b antagomir (αlet-7b), TLR7 siRNA, or scrambled RNA (scRNA) controls. At 48 h of withdrawal, slices were assessed for cell death by PI uptake. Additional slices were treated with the TLR7 antagonist CMPD2 (50 nM) throughout ethanol exposure and withdrawal. Ethanol withdrawal (EtOH WD) caused cell death evidenced by PI uptake. The let-7b antagomir blocked EtOH WD-induced cell death, returning PI uptake to scRNA control and αlet-7b alone levels. Similarly, TLR7 inhibition by siTLR7 returned neurodegeneration to control levels. The pharmacological blockade of TLR7 with CMPD2 likewise abolished ethanol-induced cell death. * *p* < 0.05 vs. control, *** *p* < 0.001 vs. control, *** *p* < 0.0001 vs. control, †† *p* < 0.01 vs. ethanol.

In order to directly investigate the mechanism underlying ethanol-induced neurodegeneration, we utilized a primary ex vivo organotypic brain slice culture (OBSC) model that contains both ENT and hippocampal brain regions. This model has been used previously to study neuronal death with ethanol exposure and withdrawal using propidium iodide (PI) uptake [51,52,53]. We first determined the sensitivity of primary OBSC slice cultures to TLR7 agonist IMQ-induced cell death. IMQ caused a concentration-dependent increase in neuronal cell death, with the highest concentration causing significant neurotoxicity at 72 h of treatment (Figure 6A). Next, we assessed ethanol-induced cell death. As mentioned above, our previous studies have found ethanol induced let-7b, the TLR7 agonist, in OBSC and that let-7b is released in microvesicles (MV) from glia but not human neuronal cultures [10]. Therefore, we determined if primary OBSC released miRNA Let-7b into the media during withdrawal as neurodegeneration progresses. We found that chronic ethanol exposure followed by withdrawal led to a progressive increase in microRNA Let-7b levels in media MVs at 24 and 48 h into withdrawal (Figure 6B). Similar to our findings in vivo, let-7b release and neuronal cell death both occurred during ethanol withdrawal (Figure 6C,D). To confirm the involvement of let-7b/TLR7 signaling, we inhibited secreted let-7b with the let-7 antagomir (αlet-7b) or blocked TLR7 (siTLR7 or CMPD2). The αlet-7b blocked ethanol-induced neurodegeneration (Figure 6C,D), with scramble control RNS (scRNA) transfection controls having no effect. Treatment with either CMPD2 or siTLR7, which we reported previously, reduces TLR7 mRNA by 50% [10], and it blocks ethanol-induced neurodegeneration (Figure 6C,D), further implicating let-7b/TLR7 signaling. Together, these findings indicate that ethanol-induced neurodegeneration involves let-7b/TLR7 signaling. 

### 2.4. TLR7 Activation Induces TRAIL-mediated Cell Death 

**Figure 7:**
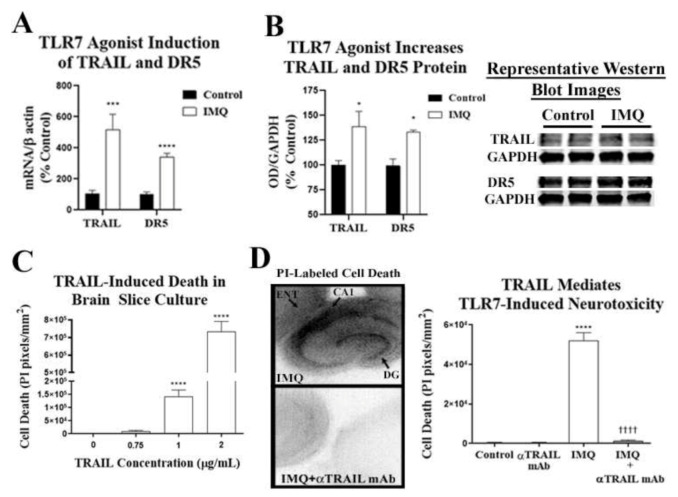
TLR7 causes neurodegeneration in hippocampal-entorhinal organotypic brain slice culture (OBSC) through TRAIL. (**A**) OBSCs were treated with the TLR7 agonist imiquimod (IMQ, 20 µM) for 16 h (20 µM) to assess for gene induction of TRAIL and its death receptor DR5. IMQ (20 µM, 16 h) caused a 3-fold induction of TRAIL mRNA in OBSC (3.5 ±.3 vs. 1.0 ± 0.9-fold TRAIL mRNA, IMQ vs. control, ** *p* < 0.01) and a 3.4-fold induction of DR5 mRNA in OBSC (3.4 ± 0.3 vs. 1.0 ± 0.1-fold DR5 mRNA, IMQ vs. control, ** *p* < 0.01). (**B**) Western blot found increased protein levels of TRAIL (≈40%) and DR5 (≈33%) with IMQ (10 µM) (**C**) OBSCs were treated with multiple concentrations of recombinant TRAIL protein and assessed for neurodegeneration. Cell death was measured by propidium iodine (PI) uptake. TRAIL caused a concentration-dependent increase in PI-labeled cell death in OBSC, which reached statistical significance at 1 µg/mL. (**D**) OBSC was treated with IMQ for 72 h ± the TRAIL monoclonal neutralizing antibody (αTRAIL mAb, 1 µg/mL) and assessed for cell death by PI uptake. IMQ caused a significant increase in PI-labeled cell death (5.2 × 10^4^ ± 4.2 × 10^3^ vs. 449 ± 260 pixels/mm^2^), which was completely blocked by TRAIL-neutralizing antibody. ANOVA: F3.35 = 139.9, * p < 0.05 vs. control, **** *p* < 0.0001 vs. control, †††† *p* < 0.0001 vs. IMQ alone, Sidak’s multiple comparisons test. *n* = 8 slices per group. Fluorescent images were converted to black and white images for ease of visualization.

Although TLR7 causes neuronal cell death, the cellular and molecular mechanism has yet to be elucidated. Since TLR7-induced neuronal death involves caspase-3 activation, we investigated if apoptotic death receptor signaling is involved. TLR7 activation in peripheral plasmacytoid dendritic cells induces expression of the death receptor ligand TRAIL/TNFSF10 and apoptosis [30], and we recently reported induction of TRAIL-associated death-inducing signaling complex (DISC) mediators in the human AUD hippocampus [20]. Therefore, we hypothesized that neuronal death due to TLR7 would involve TRAIL signaling. First, we determined whether IMQ-TLR7 stimulation with its agonist IMQ induces the expression of TRAIL and its receptor, death receptor 5 (DR5). IMQ caused a 5-fold induction of gene expression of TRAIL and a 3-fold induction of the TRAIL death receptor TRAIL-R2/DR5 (Figure 7A). Likewise, Western blot found that IMQ increased protein levels of TRAIL (≈40%) and DR5 (≈33%) (Figure 7B). Next, we determined if TRAIL induced cell death in primary OBSCs using recombinant TRAIL protein. We found a concentration-dependent increase in PI-labeled neuronal cell death staining of several fold at 1 µg/mL and over 10-fold at 2 µg/mL (42 and 84 nM respectively, Figure 7B). Additionally, in mouse cortex and hippocampus, TRAIL+IHC was primarily localized in neurons, although there may be expression in some glia (Figure S7). 

To determine whether TRAIL is involved in TLR7-induced neurodegeneration, we next assessed the efficacy of a TRAIL monoclonal antibody on IMQ-induced TLR7-mediated cell death in OBSCs. IMQ (20 µM) caused robust cell death throughout hippocampal and cortical regions of the slice culture, which was almost completely abolished by the TRAIL monoclonal antibody (Figure 7C). Different concentrations of the TRAIL antibody were tested, with 1 µg/mL showing the greatest efficacy. Thus, TRAIL inhibition prevents neuronal cell death due to TLR7 activation, implicating TRAIL-induced apoptosis as a mediator of TLR7-induced cell death. 

### 2.5. TRAIL Mediates Ethanol-Induced Cell Death in Primary Ex Vivo Brain Slice Culture 

**Figure 8:**
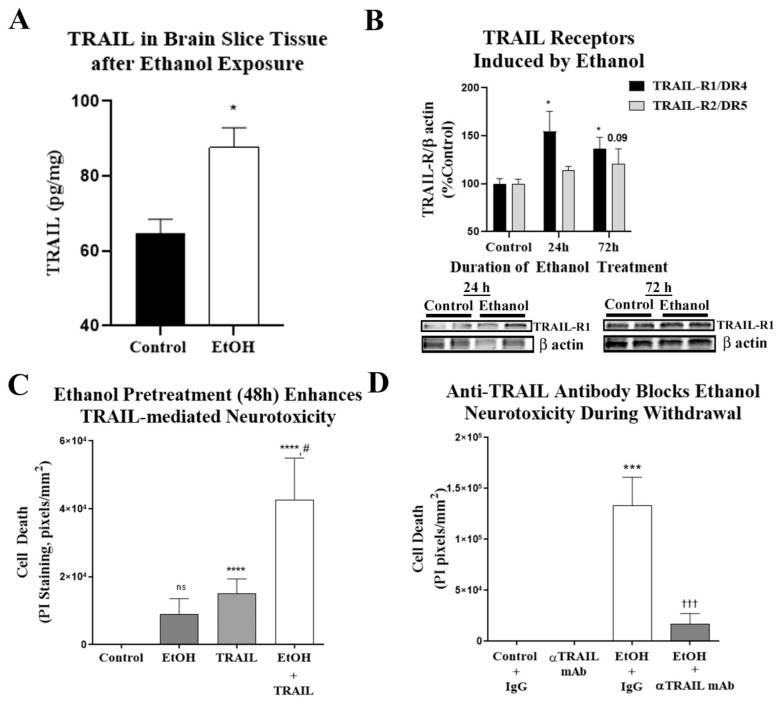
Ethanol-induced neurodegeneration in primary organotypic brain slice culture involves TRAIL. (**A**) Organotypic brain slice cultures (OBSCs) were treated with ethanol (100 mM) for 4 days. TRAIL was measured by ELISA 16 h into withdrawal prior to onset of neuronal cell death. Ethanol caused a 36% increase in TRAIL in slice tissue. *n* = 9 control, 3 ethanol slices. (**B**) OBSCs were treated with ethanol (100 mM) for 24–72 h. TRAIL-R1/DR4 and TRAIL R2/DR5 were measured by Western blot. Ethanol caused a 1.55-fold increase in TRAIL-R1 protein levels at 24 h and a 1.66-fold increase at 72 h (* *p* < 0.05). Ethanol caused a trend toward a 1.2-fold increase in TRAIL-R2 protein levels at 72 h (*p* = 0.09, *n* = 9 control and 3 ethanol). Images of blots at 72 h of EtOH treatment are shown. (**C**,**D**) The ability of ethanol pretreatment to enhance TRAIL neurotoxicity was assessed. OBSCs were treated with ethanol for 48 h, which was followed by addition of recombinant TRAIL (0.75 µg/mL) for 24 h. Neurodegeneration was assessed by propidium iodide (PI) uptake. (**C**) Ethanol and TRAIL alone caused a moderate amount of neurodegeneration. However, ethanol pretreatment caused a robust, synergistic enhancement in neurodegeneration due to TRAIL. ANOVA: F3.34 = 7.2, *p* = 0.0007. *** *p* < 0.001 vs. control, # *p* < 0.05 vs. TRAIL alone, Sidak’s multiple comparisons test. *n* = 9–10 slices/group (**D**) Representative image showing enhancement of TRAIL neurodegeneration by ethanol pretreatment. (**E**) The role of TRAIL in ethanol-induced neurodegeneration was assessed using the TRAIL monoclonal antibody (αTRAIL mAb). OBSC was treated with ethanol (100 mM) for 72 h followed by ethanol withdrawal (EW) for 24 h ± rat αTRAIL mAb (0.5 µg/mL) or rat immunoglobulin G (IgG) isotype control. EW caused significant neurodegeneration, which was completely abolished by αTRAIL mAb. ANOVA: F4.42 = 9.3, *p* < 0.0001. **** *p* < 0.0001 vs. IgG alone control, ††† *p* < 0.001 vs. EW + IgG, Sidak’s multiple comparisons test. *n* = 7–16 slices per group. (**F**) Representative images showing inhibition of EW-induced neurodegeneration by αTRAIL mAb. Fluorescent PI images were converted to black and white images for ease of visualization.

Since we observed a clear role for TLR7 in ethanol-induced neuronal death, and that TRAIL blockade prevents TLR7-induced neuronal death, we then asked if TRAIL mediates ethanol-induced neurodegeneration. First, we determined the ability of ethanol to induce the expression of TRAIL receptors and the impact on TRAIL-induced neurodegeneration. Chronic ethanol (4 days) exposure of primary OBSCs increased TRAIL protein by 35% (Figure 8A). Ethanol also increased the TRAIL death receptor protein expression, TRAIL-R1/DR4, by ≈1.5-fold (*p* < 0.05; Figure 8B). To determine if the induction of DR4 and DR5 by ethanol enhances TRAIL-induced cell death, OBSCs were treated with ethanol for 48 h, followed by exposure to recombinant TRAIL protein at a concentration we found to have moderate neurotoxicity (0.75 µg/mL, see Figure 7B). TRAIL alone caused a moderate but significant increase in PI-labeled dying cells (Figure 8C,D) that was significantly potentiated by ethanol pretreatment cell death (Figure 8C,D). Thus, ethanol induces the expression of functional active TRAIL death receptors, sensitizing neurons to TRAIL-mediated neurotoxicity. Next, we assessed if TRAIL inhibition with the anti-TRAIL monoclonal antibody (αTRAIL mAb) would block ethanol-induced neurodegeneration, as it did TLR7-induced neurodegeneration. Brain slice cultures exposed to ethanol for 4 days followed by withdrawal showed increased PI-labeled cell death that was significantly reduced by αTRAIL mAb (1 µg/mL), reducing cell death to near baseline (Figure 8D). Therefore, these studies find that ethanol causes neuronal cell death via let-7/TLR7 signaling, which induces TRAIL/DR-mediated cell death. TRAIL inhibition is effective at preventing neurodegeneration due to both ethanol and TLR7 agonists. 

## 3. Discussion

**Figure 9:**
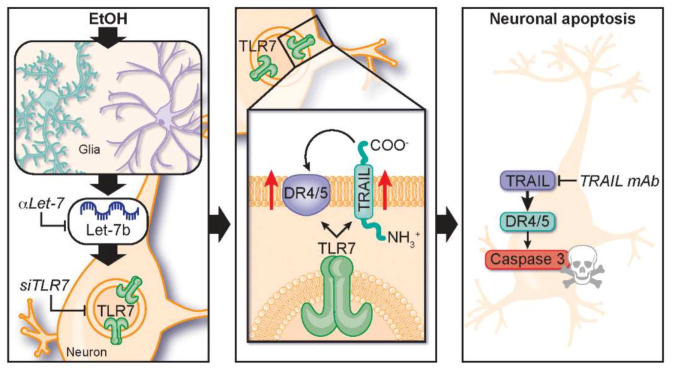
Schematic summarizing findings of let-7/TLR7/TRAIL induced neurodegeneration by ethanol. **Left panel**: Ethanol causes induction of both let-7b and its receptor TLR7. **Middle panel**: TLR7 induces expressions of the death receptor ligand TRAIL and its receptors. **Right panel**: TRAIL induces neuronal death through the induction of caspase cascades. Blocking either let-7b, TLR7, or TRAIL with the let-7 inhibitor, TLR7 siRNA, or a TRAIL neutralizing antibody respectively prevent ethanol-induced neurodegeneration.

The activation of neuroimmune signaling is associated with neurodegeneration across multiple neurological conditions. Emerging studies are finding that TLRs and endogenous TLR ligands contribute to neurodegeneration [54] and addiction [49] through the induction of complex innate immune signaling cascades. We report here that let-7b/TLR7 signaling is increased in human AUD along with apoptotic neuronal cell death. Binge levels of ethanol exposure in vivo and ex vivo each induced the expression of neuronal and microglial TLR7, increased expression of the TLR7 endogenous ligand let-7b, and enhanced TLR7-mediated pro-inflammatory cytokine and cell death responses. Ethanol-induced neuronal death was blocked by let-7 antagomirs, siRNA knockdown of TLR7, and pharmacological blockade of TLR7, implicating let-7b/TLR7-signaling in ethanol-induced neurodegeneration. TRAIL played a key role in this response, with both IMQ and ethanol increasing TRAIL and its receptors and TRAIL-neutralizing antibodies blocking neuronal cell death in both settings (Figure 9). Together, these findings implicate TRAIL as a key mediator of neuronal death involving TLR7 activation and/or ethanol. Since the pattern of neuroimmune activation in AUD is shared in other degenerative diseases, TRAIL may represent an important mediator of neuronal death in other conditions involving TLR7 activation such as viral infection [10,11,12] or Alzheimer’s disease (AD) [10,11,12,17,55,56], and it may represent a promising therapeutic target.

TRAIL and its receptors are a subset of the tumor necrosis factor receptor superfamily (TNFRSF), which contains a unique protein sequence known as the death domain (DD). DRs can induce pro-inflammatory cytokine signaling and/or initiate a death-inducing signaling complex (DISC) assembly, leading to the caspase cascades that lead to apoptotic cell death. TRAIL and its associated DRs have been historically studied in cancer and peripheral immune signaling. However, studies suggest that TRAIL signaling could play a role in AD, multiple sclerosis, mild cognitive impairment, ischemic stroke, and epilepsy [26,28,57,58,59]. TRAIL expression in healthy human brain is very low [60,61] with modest expression on glia. However, consistent with our finding of ethanol increasing neuronal TRAIL, in AD, TRAIL expression is increased in neurons [62]. Human brain tissue slices from patients with temporal lobe epilepsy also show extensive TRAIL-induced propidium iodide and cleaved caspase-3-marked neuronal apoptosis [28]. Thus, TRAIL may be a common mediator of neuronal death in several disease states.

The TRAIL-DR caspase cascade to apoptosis mechanism of neuronal death may differ from glutamate excitotoxicity necroptosis degeneration [63]. Excitotoxicity can cause necroptosis and necrosis to propagate further neuroimmune activation through the release of TLR agonists such as HMGB1 [64]. Apoptosis is not typically thought to induce further pro-inflammatory responses, and our findings indicate that it can be downstream of earlier immune activation. Interestingly, microglia seem to be resistant to DR apoptosis and instead respond with pro-inflammatory gene induction [65,66]. Furthermore, astrocytes were also found to be resistant to TRAIL-induced apoptosis through TRAIL astrocytic nitric oxide [67]. TRAIL is known to cause both immune activation and cell death [25,26] and might represent an early targetable node during the progression from neuroimmune activation to neuronal death. Our work finds that TRAIL is downstream of TLR7 (Figure 9, middle panel), and future studies will investigate if other TLRs likewise induce TRAIL-mediated neurodegeneration.

TLR7 has been found to cause neuronal death in response to its endogenous agonist let-7b as well as other viral RNAs such as HIV [10,11,12]. Let-7b miRNA stimulation by TLR7 is unique apart its role as a regulator of target mRNAs [68]. In AUD, neurodegeneration occurs without viral infection, is progressive, and is associated with cognitive decline, poor decision making, and memory deficits [18,19,31,32,33,38,39,40,41,42,43]. Previous studies of postmortem human AUD brain find that let-7 isoforms increased in postmortem human AUD brain and after long-term ethanol treatments in mice [14,15]. We now report in postmortem human AUD brain, in mice, and ex vivo brain slice cultures a novel neurodegeneration pathway involving brain miRNA let-7 activation of TLR7 signaling inducing neuronal TRAIL and DR5, which triggers caspase-3-mediated neuronal cell death. In AUD, TLR7 may be a unique neuronal death signal. We and others have found that other TLRs such as TLR4 and TLR2 are also involved in AUD glial pathology [10,69,70,71]. TLR4 and TLR2 knockout mice are protected from chronic ethanol (5 weeks) induction of striatal and serum cytokines/chemokines, autophagy impairments, glial activation, NFκB activation, cortical caspase-3 activation, anxiety-like behavior, and working memory deficits [72,73,74]. These TLRs are associated with glial activation, and cultured microglia or astrocytes from TLR4 knockout mice are protected from ethanol-induced activation [74,75]. In primary cultures, microglia are required for TLR2 or TLR4 agonist-increased neuronal cell death, but the TLR7 agonist loxoribine directly increases neuronal death independent of microglia, indicating that TLR2- or TLR4-stimulated microglia are secreting neurotoxic factors [76]. We reported that ethanol causes secretion of the TLR4 agonist HMGB1 from neurons, leading to microglial activation [10,49] and that let-7b is secreted by cultured microglia but not neurons [10]. Therefore, we surmise that glial activation by TLR2 and/or TLR4 leads to their secretion of let-7b with subsequent TLR7 activation in neurons (Figure 9, left panel).

It is important to note that we have focused on let-7b, but the let-7 family of miRNAs consists of at least 12 different isoforms in humans (e.g., let-7a, let-7b, let-7c, etc.) [77,78]. Let-7b is thought to be particularly potent at TLR7, due to its GU-rich region [12,79]. However, other miRNAs, including let-7c and miR-21, have been found to bind to TLR7 and restrict neurite outgrowth or cause cell death in different settings [55,80]. We found previously that several let-7 family members are upregulated in OBSCs by ethanol [10], and that multiple let-7 family members are induced in liver in the setting of AUD hepatitis and correlate with disease severity [81]. Let-7 isoforms have been shown to be increased in the cerebrospinal fluid of patients with Alzheimer’s disease [12,17], further suggesting that let-7/TLR7/TRAIL signaling could be involved in multiple neurodegenerative disorders and represent a useful therapeutic target.

In summary, this work identifies a role for TRAIL in TLR7-mediated neuronal apoptosis and alcohol-induced neurodegeneration. This signaling pathway may be involved in other neurodegenerative disease states. Future work will utilize novel technologies and pharmaceuticals to inhibit glial-neuronal let-7/TLR7/TRAIL signaling to prevent neurodegeneration in various models of neurodegeneration.

## 4. Materials and Methods

### 4.1. Postmortem Human AUD Brain Tissue

Paraffin-embedded sections and frozen tissue of postmortem human orbitofrontal cortex (OFC) were from the New South Wales Tissue Resource Centre (NSW-TRC) in Australia. The work was approved by the University of Sydney institutional ethics committee (X11-0107) and the University of North Carolina at Chapel Hill Institutional Research Board (#20-3401) on 18 November 2020. Written informed consent was obtained from all participants antemortem. Individuals with cirrhosis or nutritional deficiencies were excluded. Alcohol use disorder diagnoses were confirmed using the Diagnostic Instrument for Brain Studies, which complies with the Diagnostic Statistical Manual of Mental Disorders [44].

### 4.2. Animals

Eight-week old male C57BL/6 mice were purchased from Jackson Laboratory. Protocols were approved by the Institutional Animal Care and Use Committee (IACUC) at the University of North Carolina at Chapel Hill and were in accordance with NIH regulations. Experiments were performed under protocol #20-231.0 approved on 31 October 2020.

### 4.3. Reagents, Antibodies and ELISAs

Anti-TLR7 (NBP2-24906)—Novus Biologicals, Littleton, CO; anti-NeuN (MAB377), anti-GAPDH (ab2302), and Fluoro-Jade B—EMD Millipore (Temecula, CA, USA); anti-p-NFκB p65 (sc-101749)—Santa Cruz Biotechnology (Dallas, TX, USA); anti-HMGB1 (ab18256), anti-glial fibrillary acidic protein (GFAP) (ab4648), anti-DR4 (ab8414), anti-DR5 (ab8416), and anti-Iba1 (ab5076)—Abcam (Cambridge, MA, USA); cleaved caspase-3 (9661L)—Cell Signaling Technology (Danvers, MA, USA); rat anti-TRAIL antibody (550320) and rat IgG2a κ isotype control (554687)—BD Biosciences, (San Jose, CA, USA); anti-human TLR7 ELISA (MBS263437)—MyBioSource (San Diego, CA, USA); anti-rat TRAIL ELISA (LS-F23243)—LifeSpan BioSciences Inc. (Seattle, WA, USA); anti-human TRAIL ELISA (DY375)—R&D Systems (Minneapolis, MN, USA). The TLR7 antagonist Compound 2 (CMPD2) [50] was provided by EMD-Serono^TM^ (Rockland, MA, USA). 

### 4.4. TUNEL Stain and Immunohistochemistry (IHC)

IHC for human brain was performed on mounted sections from the New South Wales Brain Tissue Resource Centre (NSW-BTRC). Mice were anesthetized with sodium pentobarbital and transcardially perfused with phosphate-buffered saline (PBS). Brains were excised and post-fixed in 4.0% paraformaldehyde for 48 h at 4 °C followed by 4 days of fixation in 30% sucrose solution. Coronal sections (40 µm) were cut on a sliding microtome (MICROM HM450) and stored at −20 °C in cryoprotectant (30% glycol/30% ethylene glycol). Free-floating sections were processed for immunostaining as previously described [34,82]. TUNEL stain was performed on human and mouse brain sections according to manufacturer’s instructions (Invitrogen, Click-iT^TM^ TUNEL kit, C10625) and for co-immunofluorescence (C10245). Sections were washed in PBS, and antigen retrieval was performed by incubation in Citra solution (BioGenex, Freemont, CA, USA) for 1 h at 70 °C. After blocking, slides were incubated with primary antibody at 4 °C overnight. Sections were washed and incubated with the appropriate secondary antibody (1:200, Vector Labs; Burlingame, CA, USA) for 1 h at room temperature (RT). Immunolabeling was visualized using the avidin–biotin (ABC) method (Vectastain Elite Kit, Vector Labs; Burlingame, CA, USA) with diaminobenzidine (DAB)/nickel enhancement, as we have previously reported [34,38,83]. All sections for each experiment were dipped in DAB solution simultaneously for the same amount of time (<2 min). Negative control for non-specific binding was conducted on separate sections employing the above-mentioned procedures with the exception that the primary antibody was omitted. In each region, +IR cells were measured in 5 randomly selected locations of interest per hemisphere, for a total of 10 samples per section. BioQuant Nova Advanced Image Analysis (R&D Biometric, Nashville, TN, USA) was used for cell counts. For immunofluorescence, the day after incubation with the primary antibody, sections were incubated with the appropriate fluorescently-tagged secondary antibody (1:1000, Life Technologies, Grand Island, NY, USA) for 1 h at RT as we have previously reported [83]. Secondary-only negative controls were performed without primary antibody incubation. Sections were mounted on slides and cover-slipped with Prolong anti-fade mounting medium (Thermofisher, Waltham, MA, USA).

### 4.5. Real-Time qPCR Analysis for mRNA and miRNA let-7b

Total RNA was extracted from tissue by homogenization in Trizol (Invitrogen, Carlsbad, CA, USA) and reverse transcription as described previously [84]. Primer sequences used are in Table 2. SYBR green PCR master mix (Applied Biosystems, Foster City, CA, USA) was used for real-time PCR analysis. The relative differences between control and treatment groups were expressed as the percent difference relative to controls (ΔΔCt method). Genes of interest were normalized to housekeeping genes β actin or 18S for mRNA and snRU6 for miRNA. For miRNA, TaqMan^TM^ Advanced miRNA cDNA Synthesis Kit (Thermofisher, Waltham, MA, USA) was used for reverse transcriptase as described previously [10].

### 4.6. Chronic Binge Ethanol Treatment with Assessment of TLR7 Sensitization

Mice received water or ethanol (5 g/kg/d, i.g., 25% *w*/*v*) for 10 days. The average blood alcohol concentration at 1 h after the ethanol treatment was 279 mg/dL ± 11 (*w*/*v*, *n* = 10). For ethanol pretreatment studies, twenty-four hours after the last ethanol administration, mice were injected with either saline (control) or imiquimod (IMQ, 2.5 mg/kg, i.p., Sigma-Aldrich, St. Louis, MO, USA) and sacrificed 2 h later.

### 4.7. Experimental Protocols of Primary Ex Vivo Hippocampal–Entorhinal Cortex Organotypic Brain Slice Culture

Primary organotypic brain slice culture (OBSC) was prepared according to our previously established protocols [44]. Briefly, the hippocampal–entorhinal complex was dissected from 7-day-old rats in Gey’s buffer and sectioned transversely at 375 μm. Slices were placed on 30 mm diameter membrane inserts (Millicell-CM, Millipore Corp, Temecula, CA, USA) and cultured with medium (75% MEM, 25 mM HEPES and Hank’s salts, 25% horse serum (HS), 5.5 g/l-glucose, 2 mM l-glutamine) for 7 days, followed by culture medium containing 12.5% HS for 4 days and then in culture medium containing 6% HS throughout the experiments. Serum-free N_2_-supplemented minimum essential medium (MEM) was used to mix with culture medium containing 25% HS. All cultures were maintained in a humidified 5% CO_2_ incubator at 36.5 °C for 2 weeks prior to treatment.

To assess neuronal cell death in slices treated with TLR7 agonist IMQ or after ethanol withdrawal, propidium iodide (PI) uptake was used to measure neuronal death as we have previously reported [10,85]. Briefly, PI (5 μg/mL) was added to the culture medium 24 h prior to capturing PI fluorescence images at indicated time points. The mean PI fluorescent intensity in CA fields was determined by using AxioVision v.3.1 software (Carl Zeiss, Jena, Germany). The number of fluorescent pixels was quantified using Bioquant^TM^ imaging software v.20.7 (R&D Biometric, Nashville, TN, USA). 

For ethanol treatment, slices were treated with ethanol (100 mM) for 4 days in a desiccator containing 300 mL water that was saturated with an equal concentration of ethanol to balance the evaporation of ethanol from the media. At the end of ethanol treatment, slices were removed and cultured with ethanol-free media for 24–48 h for withdrawal. Neuronal death was determined by PI uptake.

At the end of ethanol treatment slices were treated with let-7 antagomir (αlet-7b, ThermoFisher Scientific, 4464084, MIMAT0000063) or small interfering (si)RNA against TLR7 (ThermoFisher, Waltham, MA, USA) in ethanol-free media to inhibit let-7b or TLR7, respectively. Lipofectamine RNAiMAX transfection reagent was used, as we have done previously [10]. Control and ethanol withdrawal alone groups were also treated with lipofectamine reagent and either mirVana™ miRNA Inhibitor negative control (ThermoFisher, Waltham, MA, USA) or the scrambled siRNA universal negative control (Sigma, St. Louis, MO, USA) for comparison with αlet-7b or siTLR7 treatment, respectively. Neuronal death was determined by PI uptake.

For inhibition of TRAIL, slices were incubated with the anti-TRAIL antibody or control IgG after ethanol treatment. Prior to use, azide was removed from the TRAIL antibody buffer in a Zeba^TM^ Spin desalting column according to the manufacturer’s instructions (ThermoFisher, Waltham, MA, USA). Neuronal cell death was assessed using PI uptake. 

For microvesicle (MV) let-7b analysis, culture media were collected at the end of experiments as indicated and centrifuged at 300× *g* for 10 min and 6000× *g* for 10 min. Then, the supernatant was centrifuged at 21,000× *g* for 96 min to pellet MVs. miRNA let-7b in MVs was determined as reported previously [10]. 

### 4.8. Statistical Analysis

Data are expressed as mean ± SEM and statistical significance was assessed with a one-way ANOVA using GraphPad Prism^®^ v.9.0 (GraphPad Software, San Diego, CA, USA). Post-hoc analyses were performed using Sidak’s multiple comparisons test adjusted for multiple comparisons. Student *t*-tests were performed for all pre-planned orthogonal contrasts.

## Figures and Tables

**Table 1:** Demographics of alcohol use disorder (AUD) and control subjects from New South Wales Brain Tissue Bank.

DSM-5 Alcohol Classification	Age	PMI	RIN Value	Lifetime Alcohol (g)	BAC at Death (g/dL)	Cause of Death and Agonal State/Mode of Death
Control	24	43	6.2	22,000	-	Arrhythmia, Rapid
Control	43	66	7.4	13,000	ND	Pneumonia, Intermediate
Control	44	50	7.1	69,000	-	IHD, Rapid
Control	46	29	4.4	17,300	-	MI, Intermediate
Control	48	24	6.9	59,000	-	IHD, Rapid
Control	50	30	7.5	5500	ND	IHD, Rapid
Control	50	40	8.6	9000	ND	Hemopericardium, Rapid
Control	53	16	7.9	102,000	ND	Cardiomyopathy, Rapid
Control	60	28	8	0	-	IHD, Rapid
Control	62	46	8.8	5000	ND	IHD, Rapid
Mean ± SEM	48 ± 3	33 ± 3	7.28 ± 1	35 ± 13	-	-
AUD, Mild	42	41	6.9	552	0.193	CO and EtOH, Intermediate
AUD, Moderate	43.5	43.5	8	1472	0.174	Bromoxynil/EtOH, Intermediate
AUD, Remission	44	15	7.9	639	ND	IHD, Rapid
AUD, Severe	45	7.5	7.9	1799	0.297	Drowning, Intermediate
AUD, Severe	49	44	6.4	1012	0.03	IHD, Rapid
AUD, Severe	49	16	6.2	613	ND	MI, Rapid
AUD, Moderate	50	17	7.0	2453	ND	IHD, Rapid
AUD, Severe	51	27	7.3	5212	0.395	Acute Bronchitis, Intermediate
AUD, Severe	61	23.5	6.1	8052	ND	Myocarditis, Intermediate
AUD, Severe	61	59	8.3	5621	ND	IHD, Rapid
Mean ± SEM	48 ± 3	31 ± 5	7.2 ± 1	2743 ± 829	-	-

Detailed clinical data were collected for each subject as described in Methods. All subjects were male. AUD—alcohol use disorder, PMI—postmortem interval, RIN—RNA integrity number, BAC—blood alcohol content, ND—not detected, IHD—ischemic heart disease, MI—myocardial infarction, CO—carbon monoxide, EtOH—alcohol. Agonal state terminal phase durations: rapid, <1 h; intermediate, 1–24 h; long term, >24 h.

**Table 2:** List of mouse primers for real-time PCR.

Primer	Forward 5′-3′	Reverse 5′-3′
TNFα	GACCCTCACACTCAGATCATCTTCT	CCTCCACTTGGTGGTTTGCT
IL-1β	CTGGTGTGTGACGTTCCCATTA	CCGACAGCACGAGGCTTT
IL-6	GGCCTTCCCTACTTCACAAG	ATTTCCACGATTTCCCAGAG
MCP-1	ACTGAAGCCAGCTCTCTCTTCCTC	TTCCTTCTTGGGGTCAGCACAGAC
gp91^phox^	CAGGAGTTCCAAGATGCCTG	GATTGGCCTGAGATTCATCC
TLR7	GATCCTGGCCTATCTCTGACTC	CGTGTCCACATCGAAAACAC
Human IRF7	TGGTCCTGGTGAAGCTGG	GATGTCGTCATAGAGGCTGTTGG
TRAIL-R2/DR5	CTCGGTCATATCAGTGGTGC	GTTCTGTCAGGTTCCGTGTT
TRAIL	-	-
GAPDH	GTATGACTCCACTCACGGCAAA	GGTCTCGCTCCTGGAAGATG
β-actin	GCATGGGTCAGAAGGATTCCT	TCGTCCCAGTTGGTGACGAT
18S	GGCCCTGTAATTGGAATGAGTC	CCAAGATCCAACTACGAGCTT

## Data Availability

All published data will be made available upon reasonable request.

## Supplementary Materials

The following are available online at https://www.mdpi.com/1422-0067/22/5/2547/s1.
