# Supplementary material for: TRAIL Mediates Neuronal Death in AUD: A Link between Neuroinflammation and Neurodegeneration
Source: Int J Mol Sci. 2021 Mar 4;22(5):2547. doi: 10.3390/ijms22052547 (PMC7961445; doi:10.3390/ijms22052547)
Supplement: Supplementary file 1 [file ijms-22-02547-s001.zip › Supplementary Figures and legends-REVISED.docx]

**Supplementary Files**

**
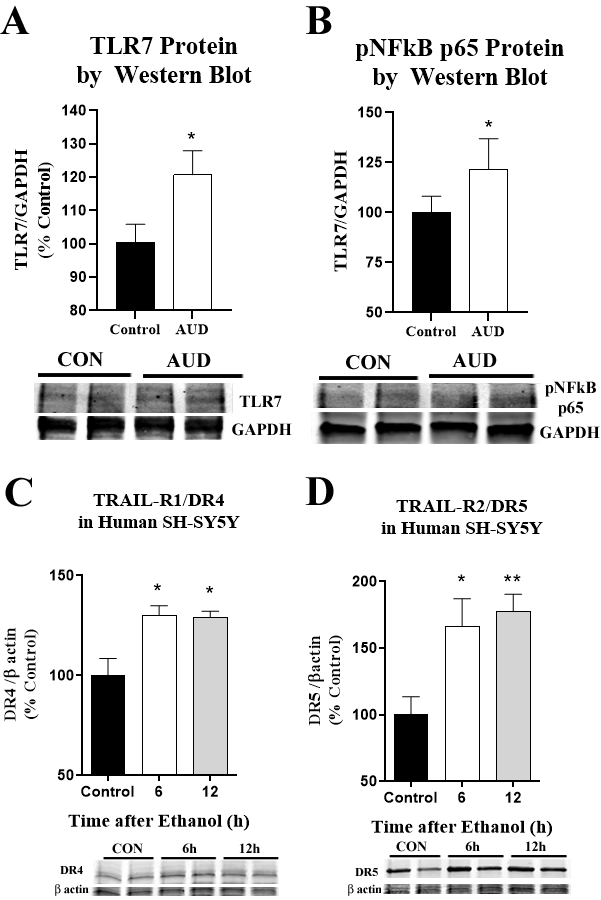
**

**Supplementary Figure 1.** **Increased TLR7 and pNFkB in human AUD OFC and ethanol induction of TRAIL death receptors in human SH-SY5Y neuronal cultures. (a)** Western blot for TLR7 on human OFC found a 20% increase in TLR7 in AUD subjects **p<*0.05 paired t-test, N=8/group. **(b)** Increased protein levels of pNFkB-p65 in human AUD subjects relative to controls, **p<*0.05. **(c-d)** SH-SY5Y were treated with ethanol (100mM) for 6-12 h. TRAIL-R1/DR4 and TRAIL-R2/DR5 were measured by western blot. **(c)** Ethanol caused a 30% increase in TRAIL-R1/DR4 at 6 and 12 h of treatment. **(d)** Ethanol caused a slight increase in TRAIL-R2/DR5 at 6 and 12 h, reaching a 12% increase at 12h. ***p*<0.01

**
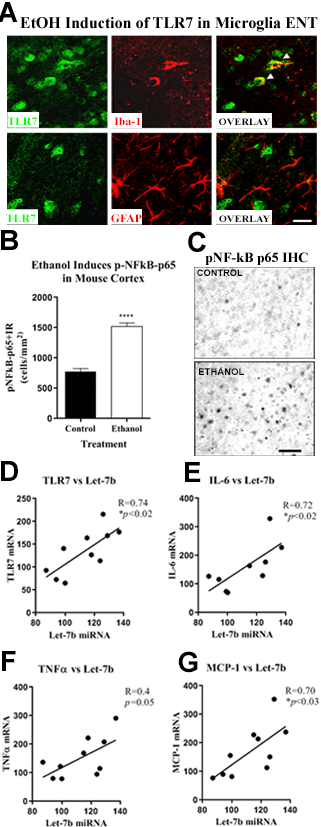
**

**Supplementary Figure 2.** **Ethanol activates NFκB and proinflammatory cytokines that correlate with miRNA let-7b expression.** C57BL/6 mice were treated with ethanol (5g/kg, i.g.) once daily for 10 days. Mice were sacrificed 24 h after the last administration and assessed for TLR7 localization in microglia and astrocytes, NFκB activation (IHC), miRNA let-7b expression and innate immune gene induction by RT-PCR. **(a)** Co-immunofluorescence of TLR7 with Iba-1+ microglia but not GFAP+ astrocytes. **(b)** Representative images showing increased p-NFκB-p65+IR cells are included. Scale bar = 50µm. **(c)** Ethanol increased phosphorylation of NFκB-p65 by 1.98-fold in the entorhinal cortex (**p<0.01, N=10 per group). **(d-g)** Let-7b expression correlated positively with multiple pro-inflammatory genes across control and ethanol-treated subjects. Let-7b correlated positively with **(d)** its receptor, TLR7, R=0.74, *p<0.02; **(e)** IL-6, R=0.72, *p<0.02; **(f)** TNFα, R=0.4, *p=*0.05; and **(g)** MCP-1, R=0.70, *p<0.03. N=5 mice/group.

**
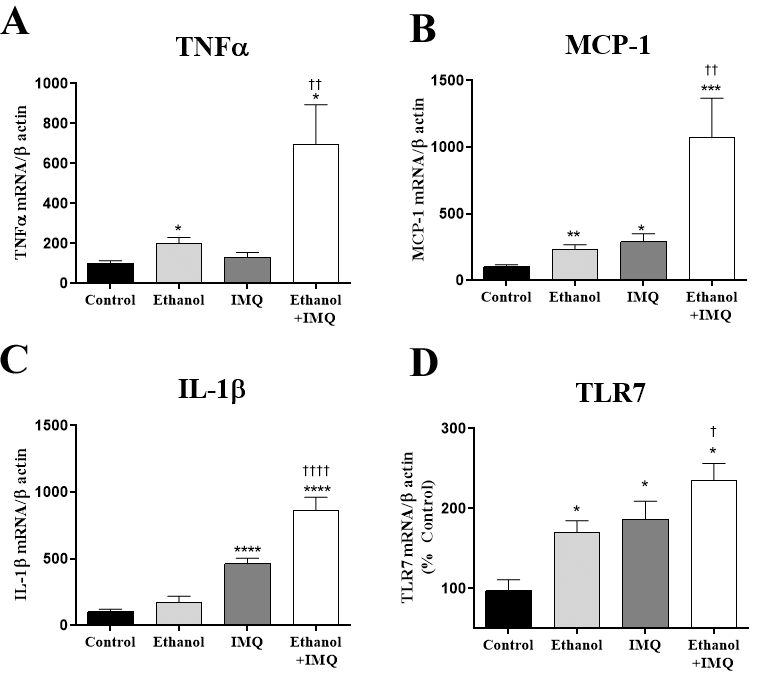
**

**Supplementary Figure 3.** **Chronic binge ethanol sensitizes TLR7 proinflammatory signaling. (a)** Experimental design of ethanol sensitization of TLR7 activation. C57BL/6 mice were treated with binge ethanol (5g/kg, i.g.) once daily for 10 days. Twenty-four h after the last administration of water or ethanol, mice were given one injection of the TLR7 agonist imiquimod (IMQ) or vehicle control. Mice were sacrificed 2 h after IMQ and whole brain assessed for innate immune gene induction by RT-PCR. **(b)** Ethanol treatment increased gene expression of TNFα 2-fold. IMQ alone had no effect on TNFα gene expression. Ethanol pre-treatment followed by acute IMQ caused a synergistic induction of TNFα (7-fold). ANOVA: F_3,16_=7.516, p=0.0023. **(c)** Both ethanol and IMQ caused moderate increases in MCP-1 mRNA, 2.4-fold and 2.9-fold, respectively. ANOVA: F_3,15_=10.46, p=0.0006. **(d)** IMQ caused a 4.6-fold induction of IL-1β mRNA. Ethanol alone did not enhance IL-1β expression (1.7-fold, p=0.20). However, ethanol pre-treatment enhanced IL-1β gene induction by IMQ to 8.6-fold of control levels (1.9-fold greater than IMQ alone). ANOVA: F_3,16_=33.86, p<0.0001 **(e)** Ethanol and IMQ alone caused a 1.7- and 1.9-fold increase in TLR7 mRNA, respectively. Pretreatment with ethanol followed by IMQ resulted in a 2.4-fold increase in TLR7 mRNA. ANOVA: F_3,16_=9.9, p=0.0006. *p<0.05, **p<0.01, ****p<0.0001 vs control, t-test, †p<0.05, ††p<0.01, †††p<0.001 vs IMQ alone, Sidak’s multiple comparison test.

**
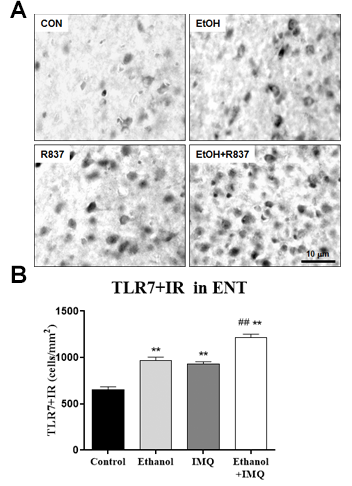
**

**Supplementary Figure 4.** **TLR7 induction by ethanol, imiquimod, and ethanol plus imiquimod.** As described in Methods, male C57BL/6 mice were treated with water or ethanol (5 g/kg, i.g., 25% ethanol w/v) daily for 10 days and injected with either saline (control) or imiquimod (R837, 2.5 mg/kg, i.p.) 24 h after the last dose of ethanol and sacrificed at 2 h after R837. The levels of brain TLR7 mRNA and protein (TLR7+IR) were measured by real-time PCR and immunohistochemistry. **(a)** Representative images of TLR7 +IR cells in the entorhinal cortex of water, ethanol, imiquimod, and ethanol+imiquimod-treated groups. Scale bar=10 µm **(b)** Ethanol and R837 alone treatment increased brain TLR7+IR cells. Ten daily doses of ethanol increased R837-induced the number of TLR7+IR cells. * *p*<0.05, ***p*<0.01, compared with the corresponding control group. $*p*<0.05, compared with the ethanol group. #*p*<0.05, ##*p*<0.01, compared with the imiquimod group.

**
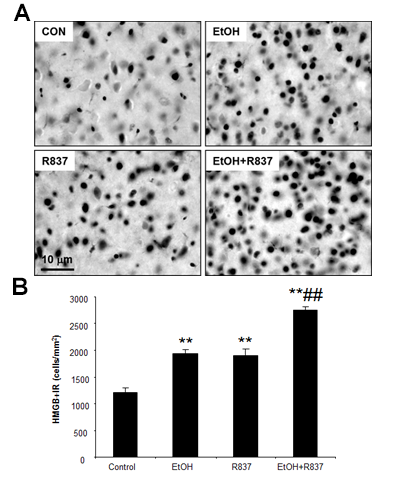
**

**Supplementary Figure 5.** **Ethanol increases HMGB1 expression and potentiates imiquimod-induced HMGB1 activation in the entorhinal cortex of C57BL/6 mice**. Brain sections from control, ethanol, imiquimod (R837) and ethanol+imiquimod treatment were immunostained with HMGB1 antibody. **(a)** Representative photomicrographs of HMGB1+IR in control, ethanol, imiquimod and ethanol+imiquimod groups. Scale bar=10 µm. ***p*<0.01, compared with the control group. **(b)** The number of HMGB1+IR cells was increased in the ENT of ethanol or imiquimod-treated group. Ethanol enhanced imiquimod-induced increase in the number of HMGB1+IR cells. ##*p*<0.01, compared with the imiquimod group.

**
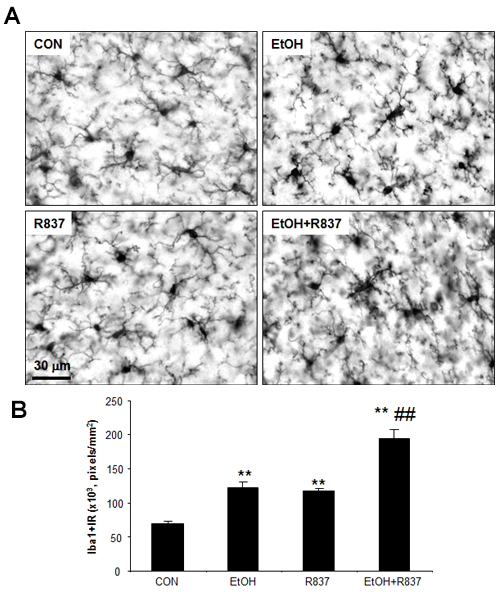
**

**Supplementary Figure 6.** **Immunohistochemical analysis of microglia.** Brain sections from control, ethanol, imiquimod (R837) and ethanol+imiquimod groups were immunostained with Iba1 antibody, a maker of microglia. **(a)** Representative photomicrographs of Iba1+IR. In the control group, microglia were in a resting, ramified morphological shape. However, in the ethanol and imiquimod-treated groups, Iba1+IR cells showed an increased cell size and irregular shape consistent with morphological changes in activated microglia. In the ethanol+imiquimod treatment showed larger cells with intensified staining, relative to ethanol or imiquimod-alone treatment groups. Scale bar=30µm. **(b)** Quantitation of Iba1+IR in the entorhinal cortex. The Iba1 immunoreactivity was significantly increased in the ethanol and imiquimod-treated group. Ethanol+imiquimod treatment showed significant increases in Iba1 immunoreactivity.

**
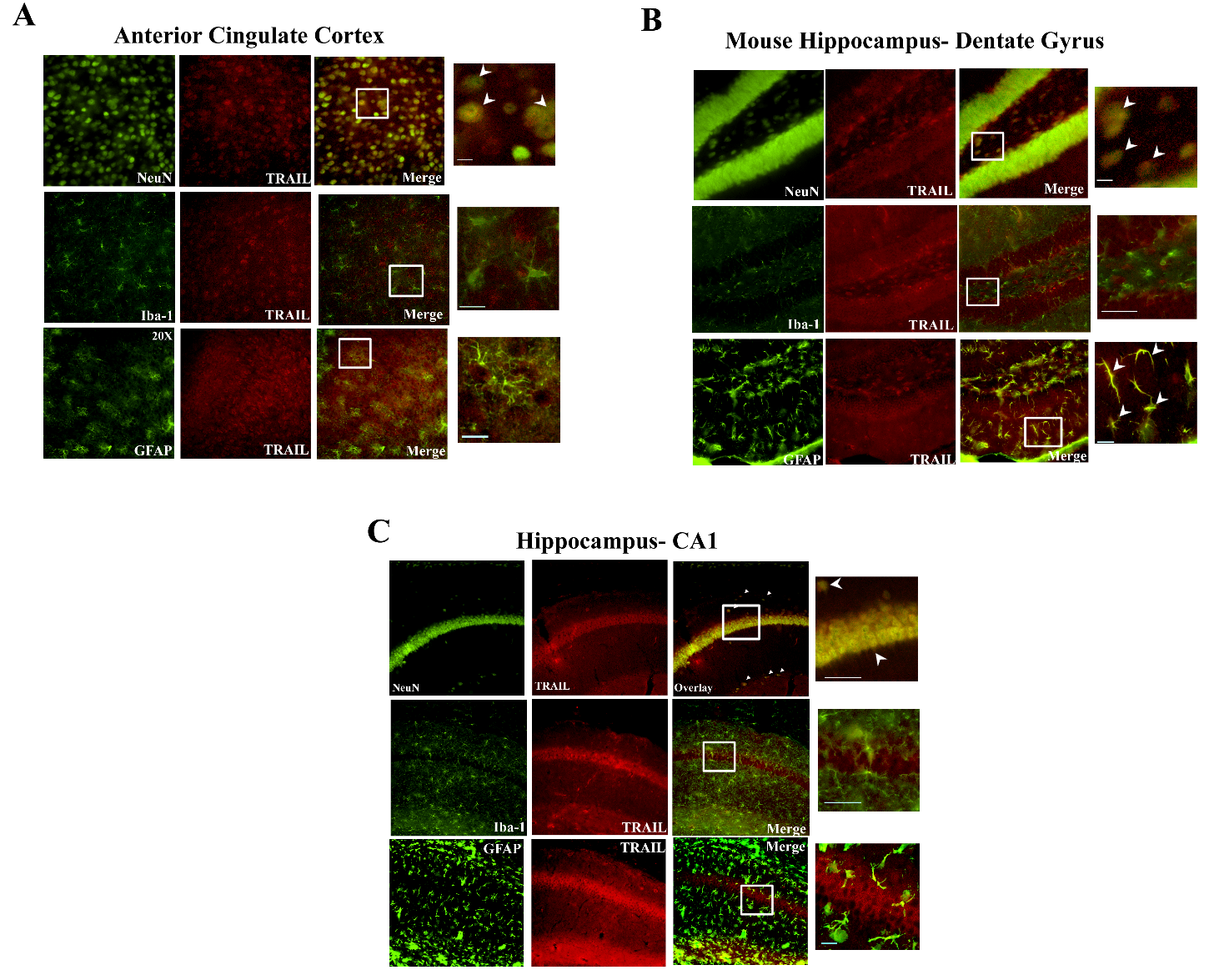
**

**Supplementary Figure 7.** **TRAIL localization to neurons and astrocytes in mouse brain. (a)** Representative images of TRAIL expression in baseline control mouse frontal cortex (anterior cingulate). TRAIL co-localized with NeuN+ neurons in cortex. **(b)** Representative images of TRAIL expression in mouse hippocampus (dentate gyrus). TRAIL co-localized with hilar neurons and astrocytes surrounding the dentate gyrus. **(c)** Representative images of TRAIL expression in mouse hippocampus (CA1). Note colocalization of TRAIL with neurons in CA1.
