# Supplementary figures and images for: TRAIL Mediates Neuronal Death in AUD: A Link between Neuroinflammation and Neurodegeneration
Source: Int J Mol Sci. 2021 Mar 4;22(5):2547. doi: 10.3390/ijms22052547 (PMC7961445; doi:10.3390/ijms22052547)

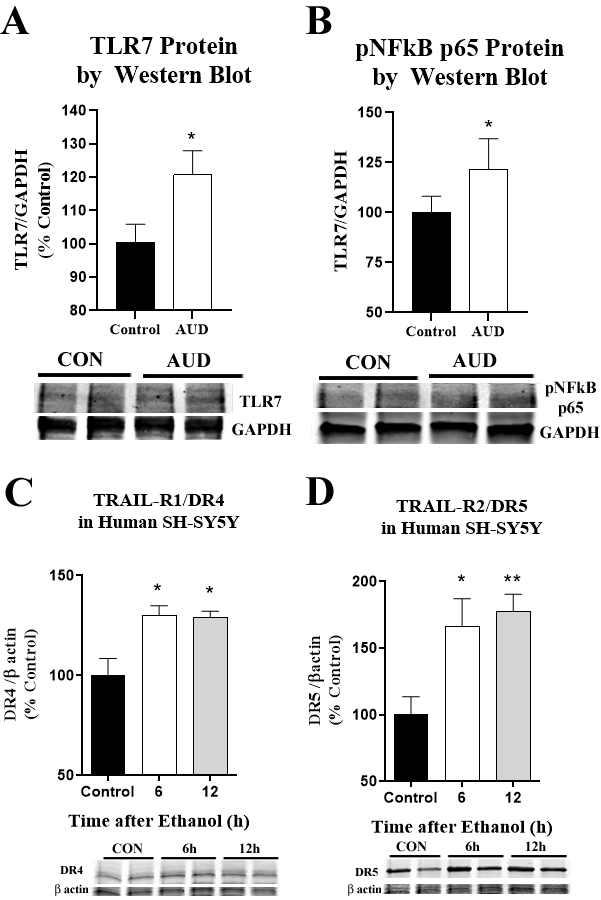

Supplement: Supplementary file 1 [file ijms-22-02547-s001.zip › S1 Figure_OFC-WBs_SH-SY5Y DRs-REVISED.tif]

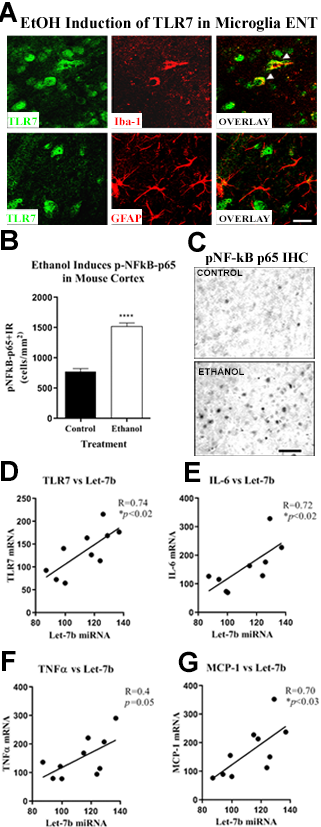

Supplement: Supplementary file 1 [file ijms-22-02547-s001.zip › S2 Figure_NfkB and cytokines_chronic ethanol-REVISED-2.tif]

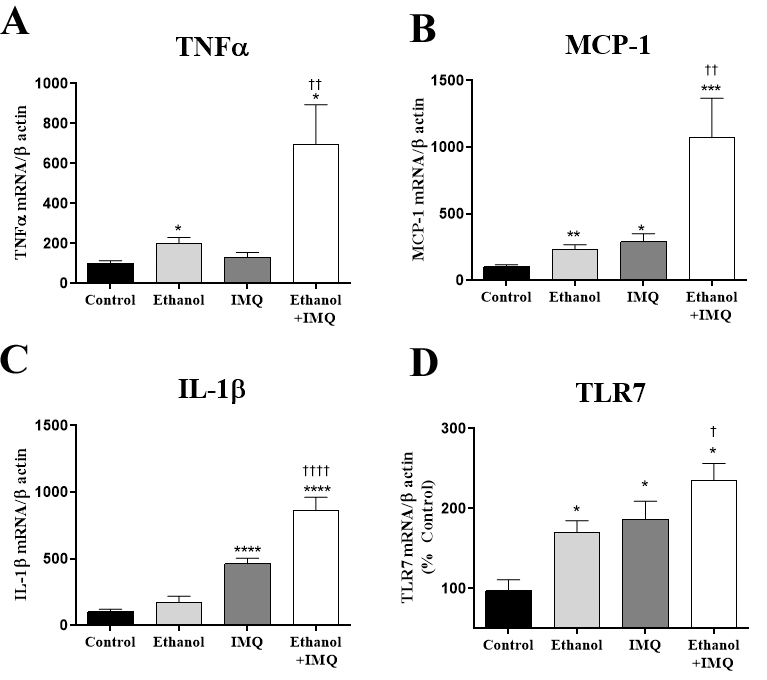

Supplement: Supplementary file 1 [file ijms-22-02547-s001.zip › S3 Figure _ethanol enhances TLR7 cytokines.tif]

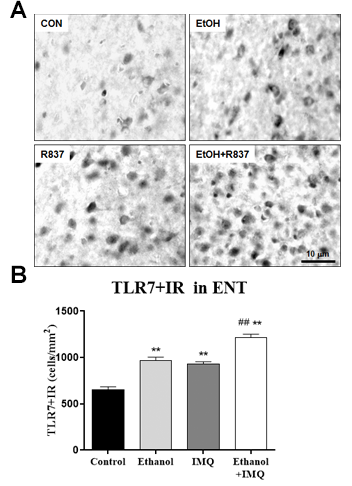

Supplement: Supplementary file 1 [file ijms-22-02547-s001.zip › S4 Figure_TLR7-REVISED.tif]

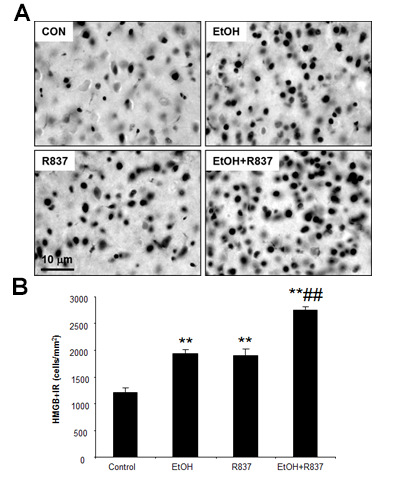

Supplement: Supplementary file 1 [file ijms-22-02547-s001.zip › S5 Figure_HMGB1-REVISED.tif]

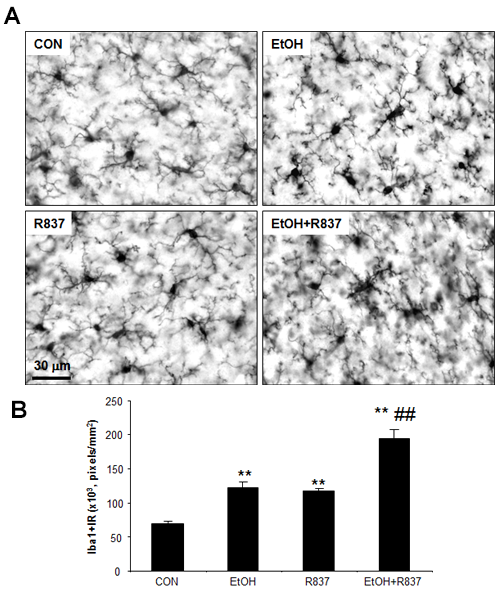

Supplement: Supplementary file 1 [file ijms-22-02547-s001.zip › S6 Figure_microglia-REVISED.tif]

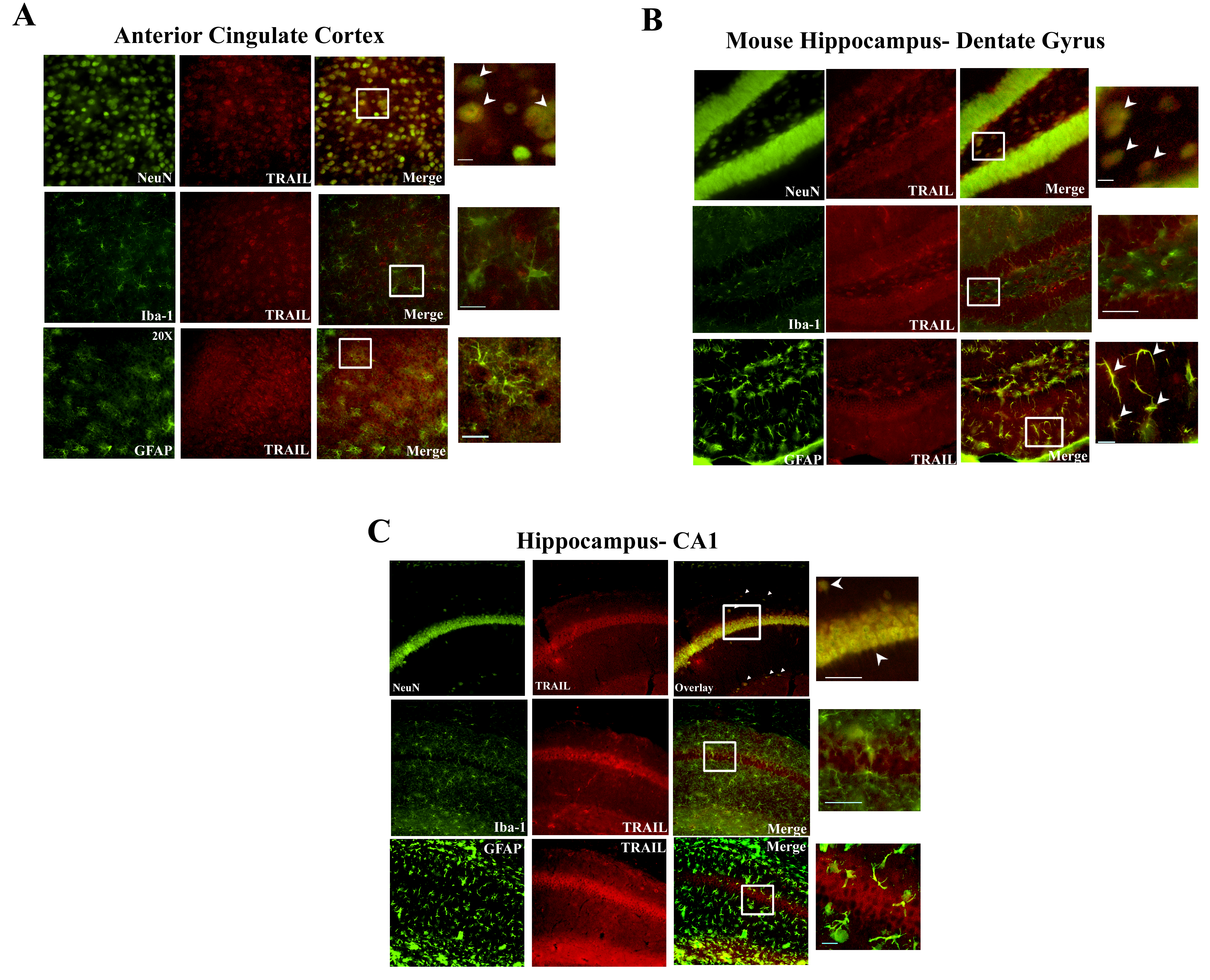

Supplement: Supplementary file 1 [file ijms-22-02547-s001.zip › S7 Figure-TRAIL and mouse brain.tif]
